# Neuroimaging for detecting covert awareness in patients with disorders of consciousness: reinforce the place of clinical feeling!

**DOI:** 10.3389/fnhum.2015.00078

**Published:** 2015-02-17

**Authors:** Lionel Pazart, Damien Gabriel, Elodie Cretin, Regis Aubry

**Affiliations:** ^1^Clinical Investigation Centre, Inserm/University Hospital, Inserm CIC 1431Besançon, France; ^2^Laboratoire de Neurosciences de Besançon EA-481, University of Franche-Comté, SFR FED 4231Besançon, France; ^3^Espace Ethique Bourgogne and Franche-ComtéBesançon, France; ^4^Pain and Palliative Medicine Department, University Hospital of BesançonBesançon, France

**Keywords:** disorders of consciousness, vegetative state, neuroimaging, electroencephalography, mental imagery, personalized medicine, ethics, medical

In the Journal, Gibson et al. (2014) report the validation of the use of two functional brain imaging techniques to complete the bedside clinical examinations for the diagnosis of and communication with patients suffering from disorders of consciousness like vegetative state (VS) or minimally conscious state (MCS). Research in neuroimaging has become a booming sector in recent years. Experimental approaches, investigating consciousness hierarchically, from a low to the highest level of cognition, and using methods such as functional Magnetic Resonance Imaging (fMRI) or ElectroEncephaloGraphy (EEG), already bring some early promising results, which have been highly publicized through the media, and thence, have undoubtedly aroused a tremendous hope in the families of people with VS or MCS. Medical teams in charge of developing new imaging tests have faced strong demand from families to apply these new tests, even if these ones were, in all likelihood, not yet completely reliable and not validated, and in spite of the fact that the ethical and social consequences were not yet fully drawn.

There is a lack of objective measures to index consciousness, as pointed out again recently by Paller and Suzuki (2014). Clinical behavioral assessment is still the gold standard and clinical studies evaluating the diagnosis performance of neuroimaging often require a control group composed of conscious healthy subjects. Nevertheless, our team (Gabriel et al., 2015) as Owen's team (Fernandez-Espejo et al., 2014) did not achieve a sensitivity of 100% for the fMRI mental imagery tasks performed on healthy volunteers. Furthermore, in most protocols, both data analysis and data interpretation are complex, ambiguous, must be taken warily, and are the subject of a much debated scientific question (Dyer, 2013; Goldfine et al., 2013). In some fMRI protocols, 41% of the subjects weren't able to be assessed, mainly because of spontaneous movement necessitating sedation before MRI (Stender et al., 2014). Works carried out with EEG, more easily affordable at bedside, have shown some limitations too (Cruse et al., 2011; Höller et al., 2013; Henriques et al., 2014). Cruse et al. (2011) noticed that 3 out of 12 healthy subjects (i.e., 25%) were unable to perform the EEG mental imagery task. According to these authors (p. 6): “Some healthy individuals might be unable to produce reliable classification, even with feedback training (so-called brain-computer interface illiterates).” In fact, this rather proves that the paradigm and/or the processing of results used are currently not accurate enough to be observed in all conscious subjects. Moreover, the replication of the same mental imagery protocol pointed out that, after correcting the experimental and statistical biases of the original study, it was impossible to observe any reliable brain activity in a group of 20 healthy volunteers (Henriques et al., 2014).

For these critics, a problem of a semiological nature arises, which is to know how to be able to assert whether or not the signs in response to a given request might be stated as specific of either a presence of consciousness or an absence of consciousness.

Whatever the brain activity obtained in response to experimental paradigms it remains extremely difficult to draw conclusions from it. As a consequence the announcement of findings is still very tricky.

The supporters of neuroimaging argue that any oriented signal is better than nothing, and constitutes a proof of an ability to be conscious. Nevertheless, the impact of false positive and false negative results is too significant to be neglected without a validation stage in order to understand exactly the meaning of the presence or the absence of an expected signal.

Instead of discard clinical signs or to be afraid of relatives' reaction in front of neuroimaging results, could we re-introduce the feelings of the close relatives and/or the medical team, regarding a potentially oriented reaction from the tested VS-patient to seek some innovative paradigms to assess the processes of consciousness by neuroimagery?

Actually, in all likelihood, it seems that the paradigms used in neuroimaging are still not adapted to the peculiar condition in which VS-patients and MCS-patients find themselves. Especially, it is quite possible that using standardized stimuli as beeps (Faugeras et al., 2012), or using some instructions which are nowhere near to be compatible with the subject's own real-life experiences (for instance, ask him/her to imagine himself/herself playing tennis, even if that has never been the case), may not induce any significant cortical or subcortical response from this patient, as those actions seem to be too distant from his former daily life. Some authors attempted the development of familiarity stimuli. Previous works with experienced volunteers seemed to enhance single-trial detectability of imagined movements when they imagine actions involving the sport or instrument with which they have experience. Nevertheless, Gibson and colleagues found no positive results for any patient in the familiar imagery task.

Perhaps we need to go further with the use of a personalized paradigm in neuroimaging for patients in VS or MCS, taking into account both their own real-life experiences and the subjective feeling of their close relatives of a kind of “presence.” Interviews of relatives or caregivers of people in VS or MCS have clearly shown the existence of some specific “signs” of a sensory nature, like some behavioral or emotional reactions, in response to a given surname, *the voice of someone*, a specific smell, music, hugs, etc. which are as many specific hints about the feeling of a kind of “presence,” or even a kind of consciousness. Very often these specific “signs” are not clinically reproducible and seem to be *distorted by affection, closeness, and subjectivity of relatives to maintain hope*. Nevertheless patient in VS or MCS could have infraclinical reactions to these types of stimuli with oriented brain activity detectable by EEG and/or fMRI.

The challenge for researchers is to transform the feelings into reproducible stimuli. The choice of stimuli used will be obtained through individual interviews, done upstream with the families and the medical teams which take care of the patients in VS or in MCS. Some examples from close relatives of patients diagnosed with VS reflect this observation of “personalized” signs of consciousness related to the perception of emotions (“I really get the impression that he reacts when he smells the aroma of pancakes”), to familiarity (“I have the feeling that he recognizes the sound of my voice” or, “… when listening to a CD featuring her children's voices, she shed some tears” or “I believe he has a reaction when he is listening to his favorite music”), or even to the patient's self-awareness (“I have the impression that he reacts to his first name”). In the last 10 years, a large number of studies have explored the reactions of patients to different types of stimuli. Emotional, linguistic, or familiar stimuli are now frequently used to assess consciousness. However, this approach is usually systematic in the sense that the consciousness of all VS patients is explored with the same stimuli, without having gathered information from relatives or health professionals about their perception of potential patient's clinical reactions. Here, we suggest adapting the task to “signs” specific to each patient. For instance, a custom protocol corresponding to the statement: “I have the impression that he/she reacts to his/her first name” could replicate those used in healthy subjects (Holeckova et al., 2006) or in patients with disorders of consciousness (Perrin et al., 2006). Such a design is easily adaptable to other types of stimuli or sensory modalities.

Thanks to the results of this “personalized” paradigm, merged with those issued from “standardized” procedures, we hope to be able to remove at least partially the doubts and the suffering of close relatives of the patients in VS or MCS. The uncertainty of that feeling of presence affects what should be undertaken by the medical profession: either increase daily sensory stimuli or prepare the close relatives to a “grieving process.” This questioning is at the heart of key ethical issues.

## Conflict of interest statement

The authors declare that the research was conducted in the absence of any commercial or financial relationships that could be construed as a potential conflict of interest.
